# Clinical Society of Maryland

**Published:** 1892-07

**Authors:** Wm. T. Watson

**Affiliations:** Secretary; 519 N. Broadway, Baltimore, Md.


### CLINICAL SOCIETY OF MARYLAND.

BALTIMORE, MD., March 18, 1892.

The 264th regular meeting was called to order by the President, Dr. Robert W. Johnson.

DR. W. S THAYER reported a case of leukæmia, and exhibited the patient. The patient, a colored man of about 30 years of age, first reported to the Johns Hopkins Hospital Sept. 15, 1890 complained of shortness of breath, swelling of feet and great swelling of abdomen. The spleen was found to be enormously enlarged, filling up the whole of the left side of the abdomen and reaching beyond the median line. Examination of the blood showed one white to four red corpuscles. There was no history of malaria. He was placed upon Fowler's solution, three minims *ter in die*, increased by one minim every other day until physiological effects appeared. In about four weeks, the proportion of white to red corpuscles was one to seventeen. He then returned home to Virginia and ceased to take medicine. He came back again Jan. 29, 1891, his blood at that time showing a proportion of one white to three red corpuscles. He was put upon arsenic, and within 19 days the leucocytosis had entirely disappeared.

May 27 he went away feeling very much better. His white corpuscles were normal and his red corpuscles numbered 4,000,000 in the cubic millimetre. His spleen, which had touched on the right Poupart's ligament, was reduced in size, extending only a hand's breadth below the costal margin. He took arsenic in increasing doses until the dose reached 12 or 13 minims *ter in die*. When physiological symptoms appeared, the dose was reduced and then started up again. He took 12 minims regularly for a matter of two months without any symptoms. Nothing was heard of the patient for 8 1-2 months. On Feb. 9th of this year he returned He said he had felt perfectly well until last Christmas. An examination revealed that his spleen was larger than ever before, legs œdematous and a proportion of one white to every nine red corpuscles. After a week's treatment in the dispensary, he was admitted to the hospital where he has been for four weeks. During this time the red corpuscles have increased from 2,700,000 to 3,400,000 and the white have diminished so that the proportion instead of being one to nine is now one to thirty-five. He has gained ten pounds. His spleen is somewhat diminished in size ; and his general condition has improved very much. His reaction to arsenic each time has been very striking.

It is in this condition with enlarged spleen, that treatment seems to do most good. Some cases are reported where there seems to have been a definite recovery.

DR. J. E. MICHAEL : Only a few years ago this case would have passed for a malarial one in the hands of the general practitioner, especially as the man comes from a malarial region. We owe a debt to Dr. Thayer for the thorough way in which he has presented the case to the Society.

DR. JAMES T. SMITH read a paper on the "The Blood in Disease."

DR. J. F. MARTINET, referring to a case mentioned by him at a previous meeting, of a woman suffering from acute mania associated with ovarian excitement, stated that the patient had been operated upon by Dr. Rohe at the State Insane Asylum. A week after the operation Dr. Rohe wrote that after she rallied from the operation her mind was perfectly clear and continued to be ever since. Previous to the operation she was one of the worst patients in the asylum. The nature of the operation, Dr. Martinet thought, was removal of the ovaries.

On motion of Dr. Michael, the following resolution was adopted: That the Clinical Society enter its protest against the proposed reduction in the appropriation for the library in the surgeon general's office at Washington, and that the President be directed to send this protest to the congressmen representing the State.

WM. T. WATSON, M. D., Secretary.
519 N. Broadway, Baltimore, Md.

